# Supplementary material for: Fluvastatin suppresses breast cancer initiation and progression via targeting CYP4Z1
Source: Commun Biol. 2026 Jan 12;9:254. doi: 10.1038/s42003-026-09532-y (PMC12910054; doi:10.1038/s42003-026-09532-y)
Supplement: Supplementary file 2 — Supplementary Information [file 42003_2026_9532_MOESM2_ESM.pdf]

Supplementary Information

Supplementary Figure 1-13 and Supplementary Table 1-3

Fluvastatin suppresses breast cancer initiation and progression via targeting CYP4Z1

Huilong Li[1,#], Ying Chen[1,#], Wanjin Shi[1], Zheng Miao[1], Yu Lu[1], Xuedan Han[1], Haitao Chen[1], Yunnan Zhang[2], Miaomiao Niu[1], Shengtao Xu[1], Hai Qin[3,*], Lufeng Zheng[1,*], Qianqian Guo[2,4,*]

[1]School of Life Science and Technology, Department of Medicinal Chemistry, School of Pharmacy, China Pharmaceutical University, 639 Longmian Road, Nanjing, Jiangsu Province 211198, China.

[2]Department of Pharmacy, The Affiliated Cancer Hospital of Zhengzhou University & Henan Cancer Hospital, Zhengzhou 450008, P. R. China.

[3]Department of Clinical Laboratory, Beijing Jishuitan Hospital Guizhou Hospital, No. 206, Sixian Street, Baiyun District, Guiyang City, Guizhou Province, China.

[4]State KeyLaboratory of Neurology and Oncology Drug Development, Nanjing, China.

[#]These authors contributed to this work equally.

[*]Correspondences: Qianqian Guo (zlyygqq4265@zzu.edu.cn), Lufeng Zheng (zhlf@cpu.edu.cn), Hai Qin (18786665889@163.com)

Supplementary Figures and Legends

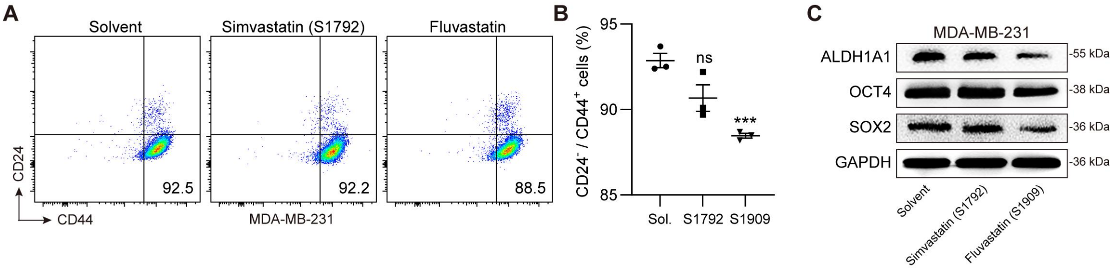

Supplementary Figure 1. Screening of CYP4Z1 inhibitors for inhibition of breast cancer cell stemness, related to Figure 1

(A–B) FACS analysis of the effects of S1909 (fluvastatin, 1 μM, dissolved in DMSO) and S1792 (simvastatin, 1 μM, dissolved in DMSO) on CD24 and CD44 expression in MDA-MB-231 cells. Each group included three biological replicates, with corresponding statistical analysis.

(C) Changes in protein abundance of stemness markers in MDA-MB-231 cells treated with S1909 (fluvastatin, 1 μM, dissolved in DMSO) and S1792 (simvastatin, 1 μM, dissolved in DMSO).

Values of $P < 0.05$ were considered statistically significant.***$P < 0.001$ vs NC group, ns indicates no significant differences from control. Error bars in the figures represent the standard error of the mean (SEM).

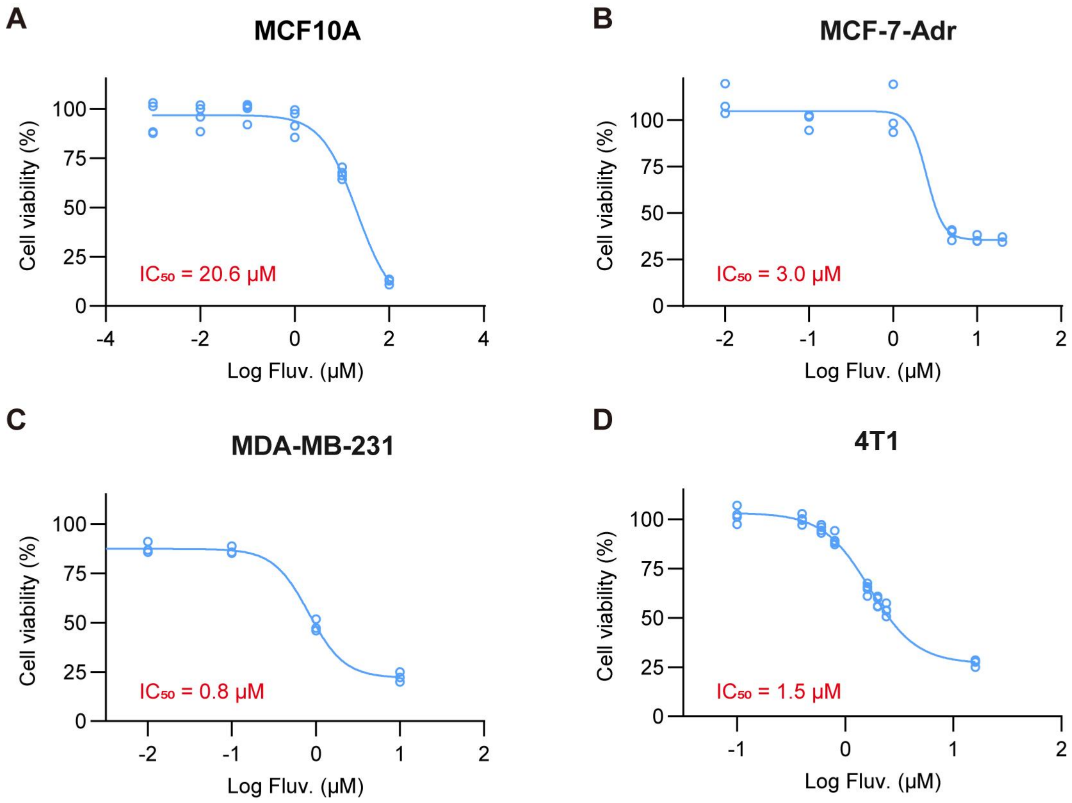

Supplementary Figure 2. The IC_{50} of fluvastatin in each cell line, related to Figure 2

(A-D) The IC_{50} of fluvastatin in each cell line was determined using CCK-8 assay. Based on the obtained IC_{50} values, the fluvastatin treatment concentrations for each cell line were established as follows: **2.4 μM** for MCF-7-Adr cells, **0.6 μM** for MDA-MB-231 cells, and **1.2 μM** for 4T1 cells.

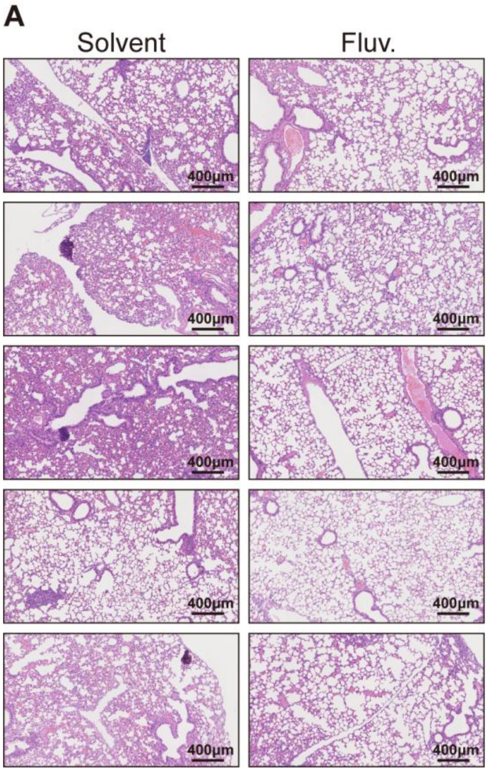

Supplementary Figure 3. The H&E staining results of the lungs of nude mice with or without fluvastatin administration, related to Figure 3

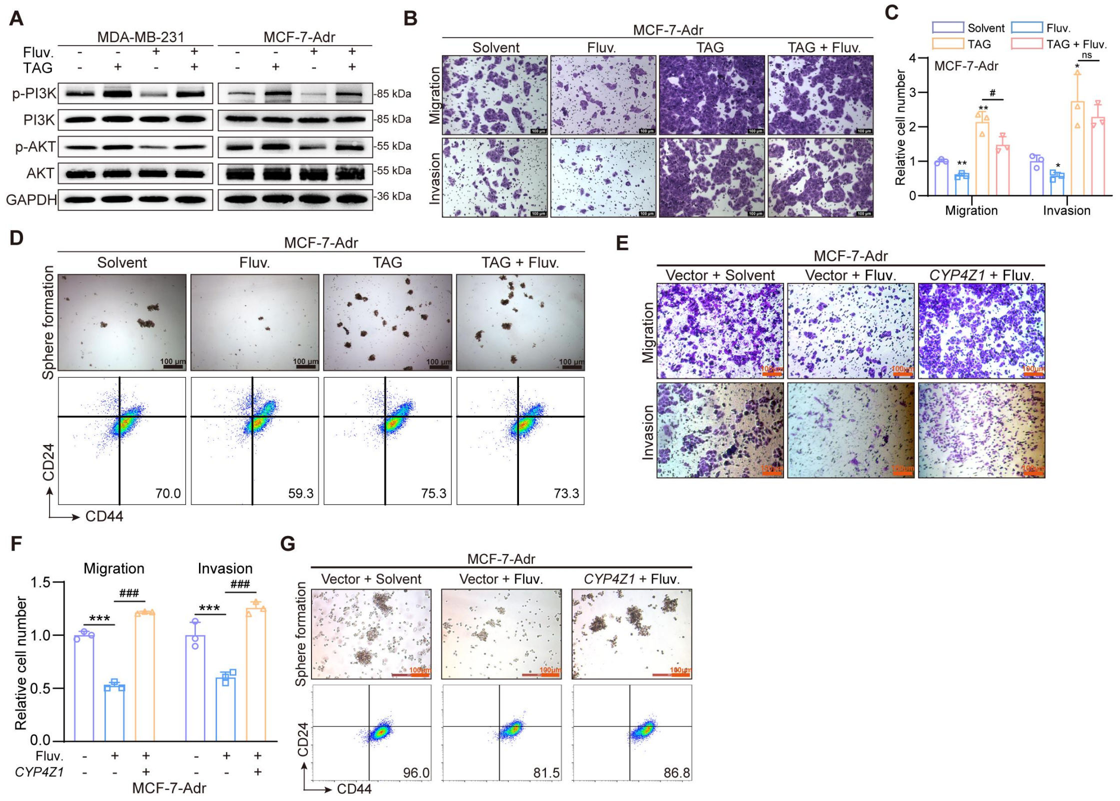

Supplementary Figure 4. Fluvastatin attenuates the stemness of breast cancer cells via targeting CYP4Z1, related to Figure 4

(A) Protein abundance changes of PI3K, p-PI3K, AKT, and p-AKT in MDA-MB-231 and MCF-7-Adr cells treated with fluvastatin (0.6 or 2.4 μM, dissolved in PBS) and TAG (300 μM, dissolved in DMSO).

(B-C) Representative images of Transwell assays in MCF-7-Adr cells and corresponding statistical analysis. Relative values were calculated as the ratio of each value to the mean value of the solvent group.

(D) Upper: Representative images of tumorsphere formation assays in MCF-7-Adr cells; **Lower:** FACS analysis of CD24 and CD44 expression in MCF-7-Adr cells.

(E-F) Representative images of Transwell assays in MCF-7-Adr cells and corresponding statistical analysis. Relative values were calculated as the ratio of each value to the mean value of the solvent group.

(G) Upper: Representative images of tumorsphere formation assays in MCF-7-Adr cells; **Lower:** FACS analysis of CD24 and CD44 expression in MCF-7-Adr cells.

Scale bar, 100 μm. Values of $P < 0.05$ were considered statistically significant.***$P < 0.001$ vs NC group. **$P < 0.01$, ***$P < 0.001$; #$P < 0.05$, ###$P < 0.001$, ns indicates no significant differences from control. Error bars in the figures represent SEM.

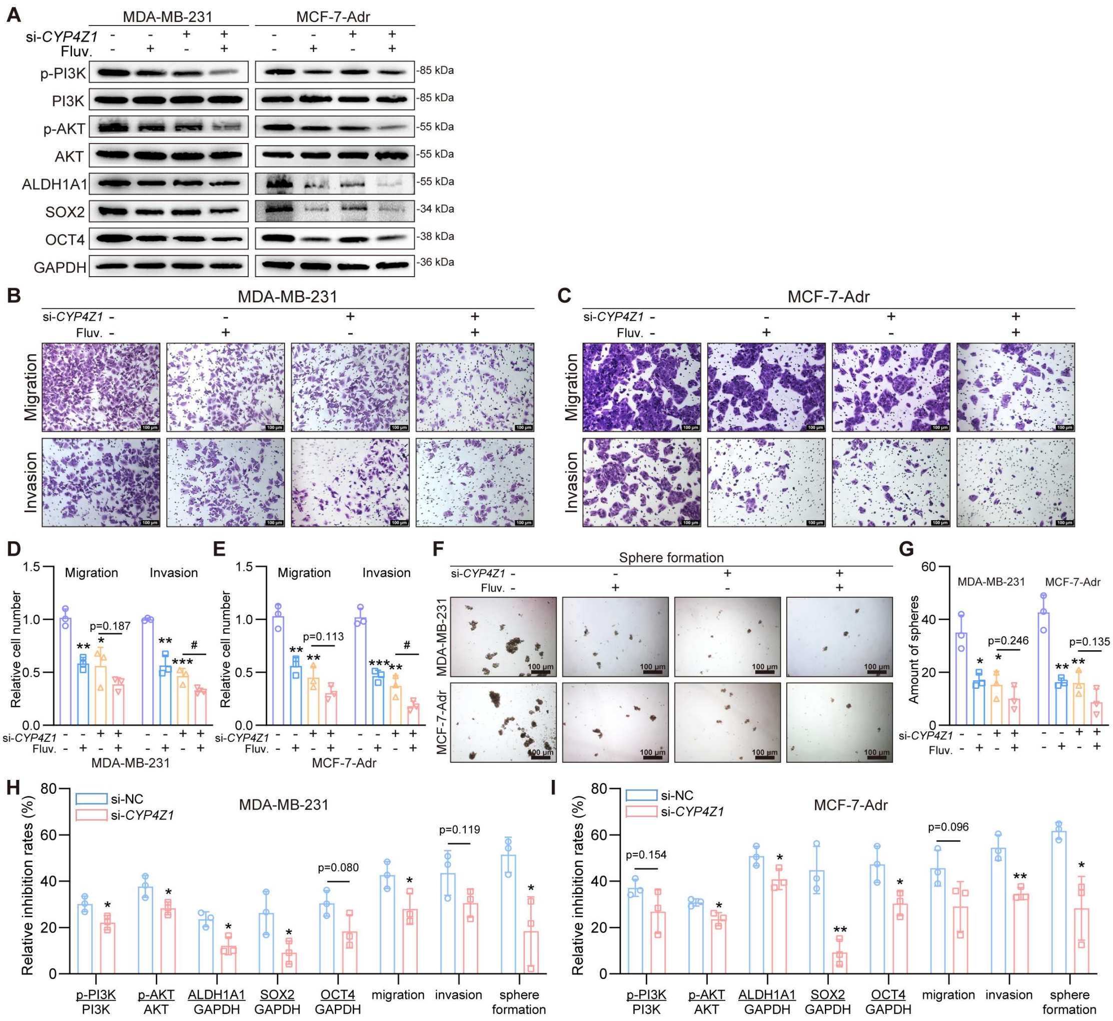

Supplementary Figure 5. Fluvastatin attenuates the stemness of breast cancer cells via targeting CYP4Z1, related to Figure 4

(A) Changes in protein abundance of stemness markers and PI3K/AKT signaling pathway components in MDA-MB-231 and MCF-7-Adr cells following CYP4Z1 knockdown and fluvastatin treatment.

(B-E) Representative images of Transwell assays in MDA-MB-231 and MCF-7-Adr cells and corresponding statistical analysis. Relative values were calculated as the ratio of each value to the mean value of the solvent group.

(F-G) Representative images of tumorsphere formation assays in MDA-MB-231 and MCF-7-Adr cells. Statistical analysis is shown, where each value represents the number of spheres in one well of a 24-well plate.

(H-I) Relative inhibition rates of fluvastatin on PI3K/AKT signaling pathway proteins, stemness marker proteins, migration, invasion, and sphere formation ability in MDA-MB-231 and MCF-7-Adr cells, measured in both the normal model and CYP4Z1 knockdown model. The relative inhibition rate was calculated as the ratio of the value

in the fluvastatin-treated group to the mean value in the solvent-treated group, within groups with the same CYP4Z1 expression background. Each dot represents one biological replicate.

Scale bar, 100 μm. Values of $P < 0.05$ were considered statistically significant.***$P < 0.001$ vs NC group. **$P < 0.01$, ***$P < 0.001$; #$P < 0.05$, ###$P < 0.001$, ns indicates no significant differences from control. Error bars in the figures represent SEM.

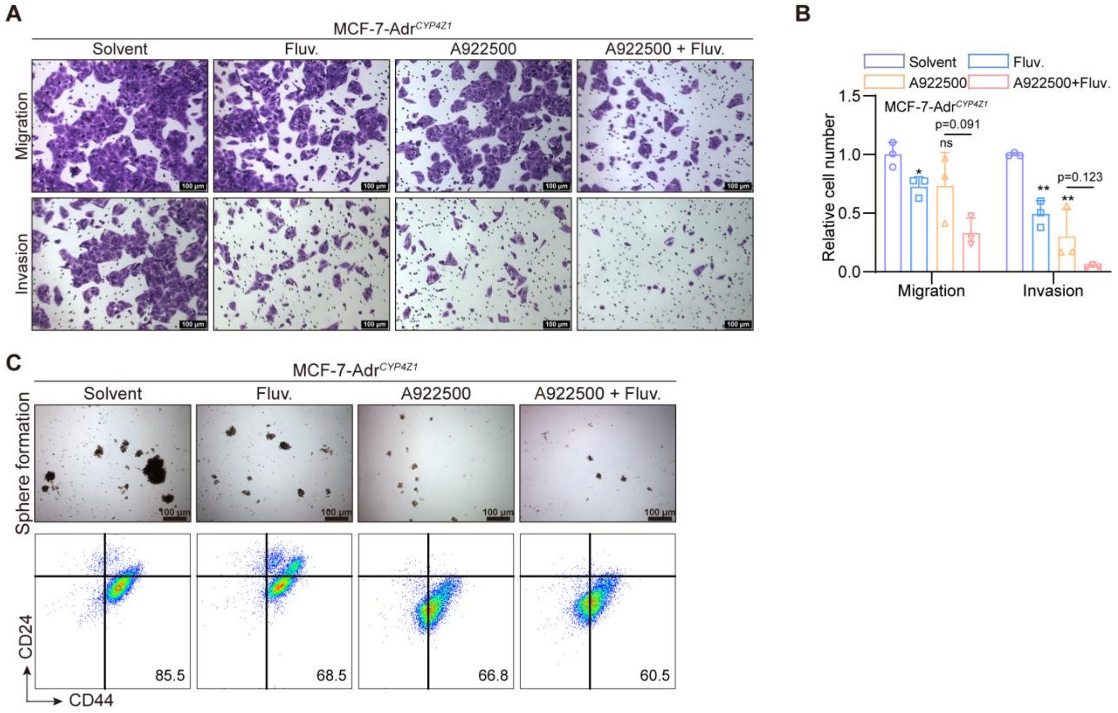

Supplementary Figure 6. Fluvastatin attenuates the stemness of breast cancer cells via targeting CYP4Z1, related to Figure 4

(A-B) Representative images of Transwell assays in MCF-7-Adr^{CYP4Z1} cells and corresponding statistical analysis. Relative values were calculated as the ratio of each value to the mean value of the solvent group.

(C) Upper: Representative images of tumorsphere formation assays in MCF-7-Adr^{CYP4Z1} cells; **Lower:** FACS analysis of CD24 and CD44 expression in MCF-7-AdrC^{YP4Z1} cells.

Scale bar, 100 μm. Values of $P < 0.05$ were considered statistically significant. **$P < 0.01$ vs NC group. Error bars in the figures represent SEM.

A

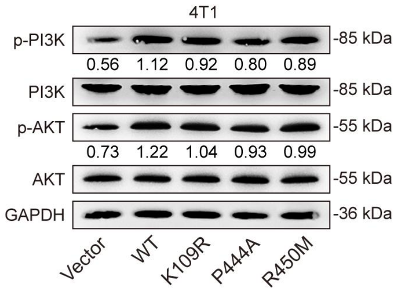

Supplementary Figure 7. Changes in protein abundance of PI3K, p-PI3K, AKT, and p-AKT in 4T1 cells overexpressing CYP4Z1 and its mutants, values represent the gray value ratio of phosphorylated protein to total protein, related to Figure 5

A

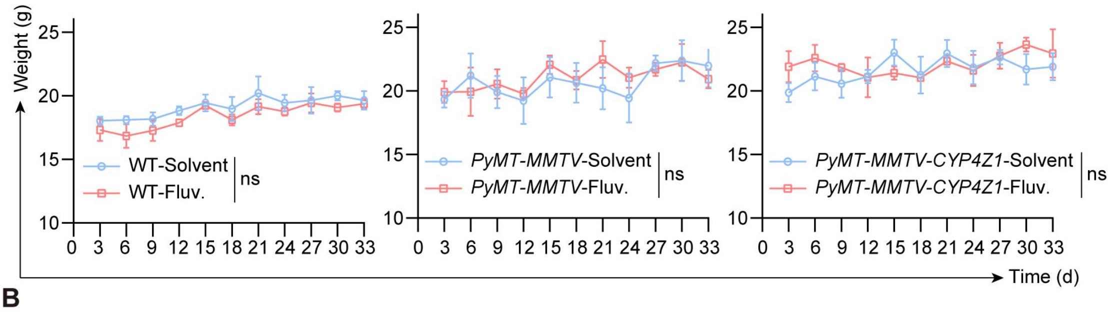

B

Items	Reference range	WT-Solvent	WT-Fluv.	*PyMT-MMTV* -Solvent	*PyMT-MMTV* -Fluv.	*PyMT-MMTV* -*CYP4Z1*-Solvent	*PyMT-MMTV* -*CYP4Z1*-Fluv.
WBC(10^9/L)	0.8-10.6	5.8	3.3	4.8	3.8	4.4	3.7
RBC(10^{12}/L)	6.5-11.5	9.54	8.7	7.75	10.24	9.91	9.28
HGB(g/L)	110-165	149	131	120	159	158	140
PLT(10^9/L)	400-1600	936	1307	694	772	1176	1250
LY(10^9/L)	0.6-8.9	5.1	2.3	3.9	3.2	3.2	3.0
GR(10^9/L)	0.23-3.6	0.6	0.9	0.8	0.6	1.0	0.6
MO(%)	0.9-18	1.4	4.2	3.3	2.3	3.6	3.5

C

Bone marrow cell	WT-Solvent	WT-Fluv.	*PyMT-MMTV* -Solvent	*PyMT-MMTV* -Fluv.	*PyMT-MMTV* -*CYP4Z1*-Solvent	*PyMT-MMTV* -*CYP4Z1*-Fluv.
LSK(%)	0.96	0.87	1.37	1.38	1.55	1.44
MP(%)	11.2	13.3	10.4	11.3	10.8	16.7
CMP(%)	40.1	34.9	25.1	30.9	42.1	27.1
GMP(%)	38.5	45.8	46.5	50.0	23.6	51.8
EMP(%)	12.0	10.8	16.2	13.4	15.9	7.31
HSC(%)	10.6	9.20	10.7	10.4	5.17	12.2

D

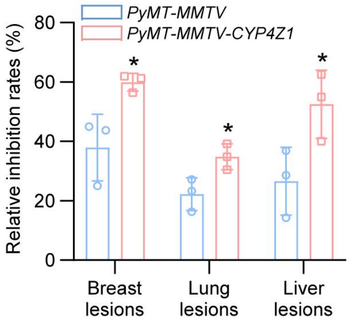

Supplementary Figure 8. Fluvastatin holds little effects on the hematopoietic capacity of mice, related to Figure 6

(A) Body weight changes of transgenic mice during fluvastatin or solvent administration.

(B) Results of blood routine analysis of transgenic mice after fluvastatin or solvent administration.

(C) Changes in the proportions of hematopoietic cells in transgenic mice after fluvastatin or solvent administration.

(D) Relative inhibition rates of fluvastatin on breast lesions, lung lesions and liver lesions ability in transgenic model. The relative inhibition rate was calculated as the

ratio of the value in the fluvastatin-treated group to the mean value in the solvent-treated group, within groups with the same CYP4Z1 expression background. Values of $P < 0.05$ were considered statistically significant. **$P < 0.01$, ns indicates no significant differences from control. Error bars in the figures represent SEM.

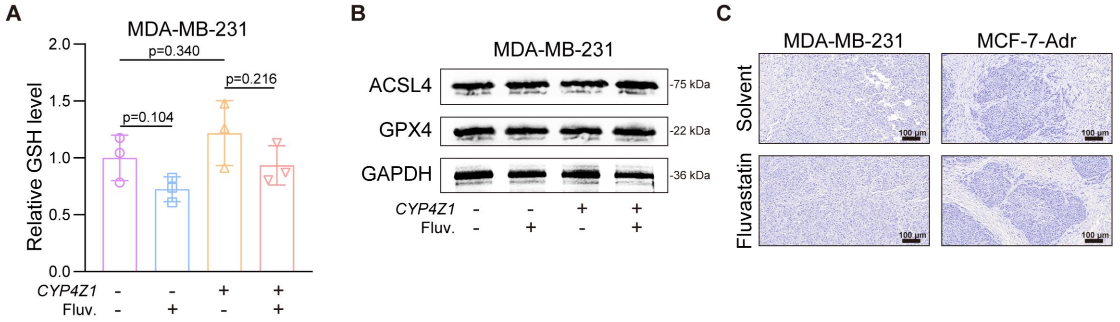

Supplementary Figure 9. Neither CYP4Z1 nor fluvastatin exerts a significant effect on ferroptosis in breast cancer, related to Figure 6

(A) Effects of CYP4Z1 manipulation and fluvastatin treatment on intracellular GSH levels in MDA-MB-231 cells.

(B) Changes in protein abundance of ACSL4 and GPX4 in MDA-MB-231 cells following CYP4Z1 overexpression and fluvastatin treatment.

(C) Prussian blue DAB-enhanced staining to detect iron deposition in xenograft tumors derived from MDA-MB-231 and MCF-7-Adr cells, treated with either solvent or fluvastatin. Scale bars, 100 μm. Error bars in the figures represent SEM.

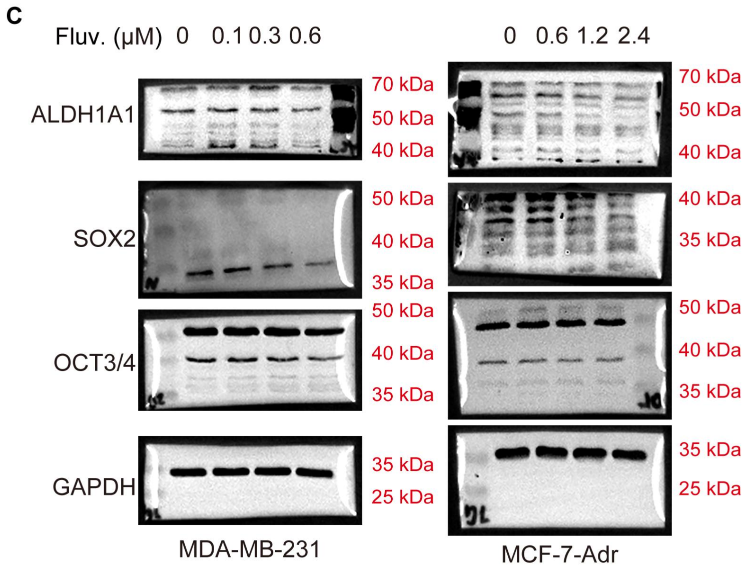

Supplementary Figure 10. Unedited western blots images, related to Figure 2

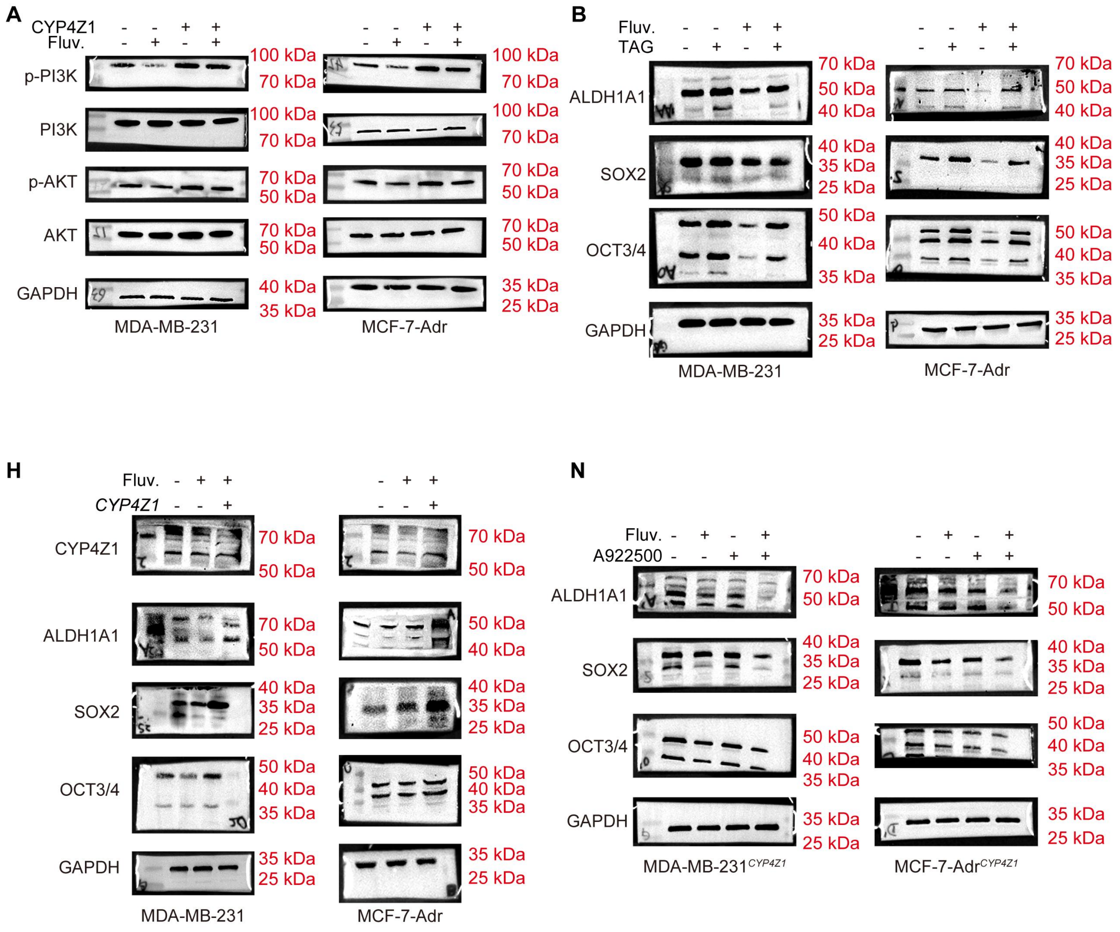

Supplementary Figure 11. Unedited western blots images, related to Figure 4

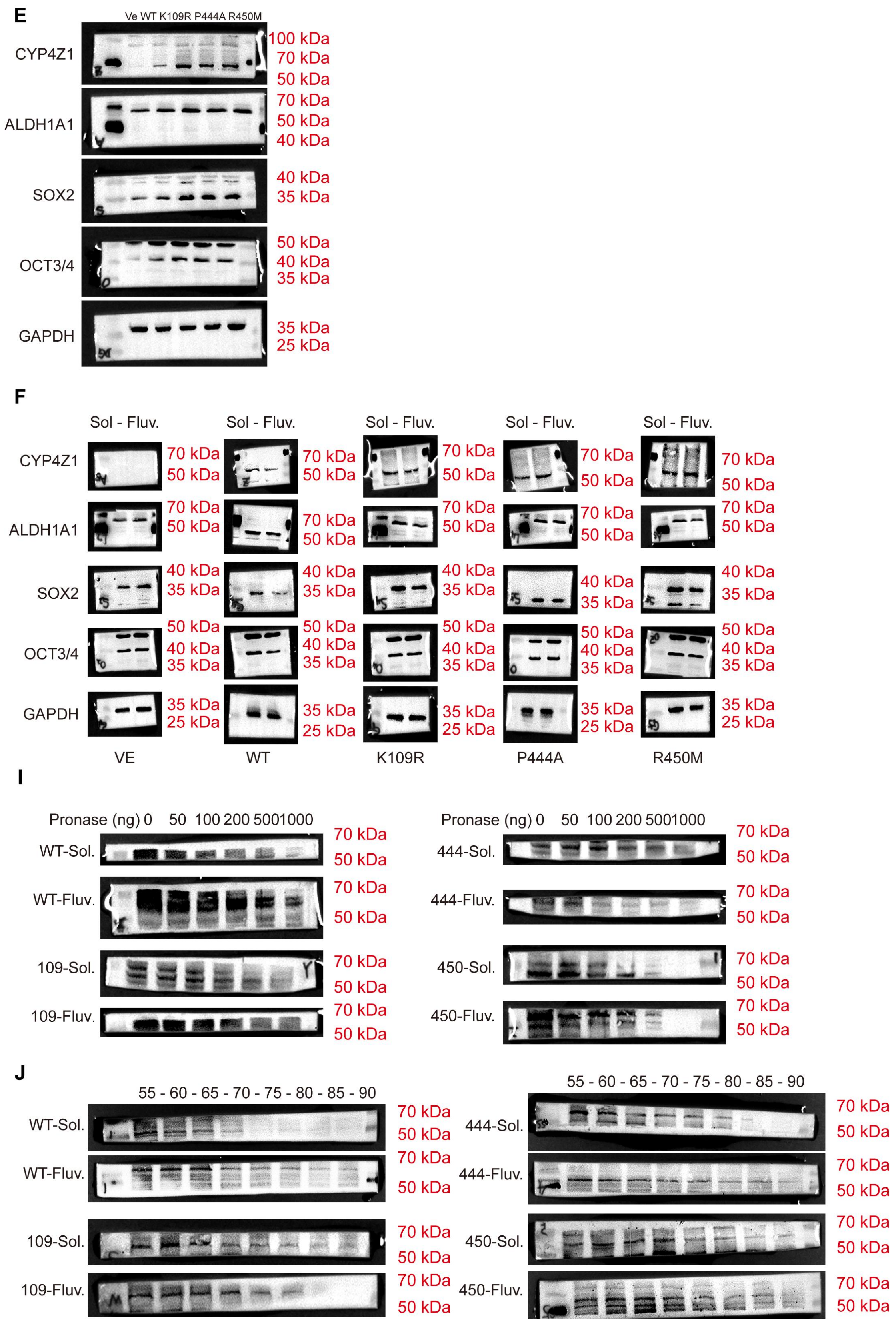

Supplementary Figure 12. Unedited western blots images, related to Figure 5

Uncropped western blot images, related to Supplementary Figure

Supplementary Figure 1

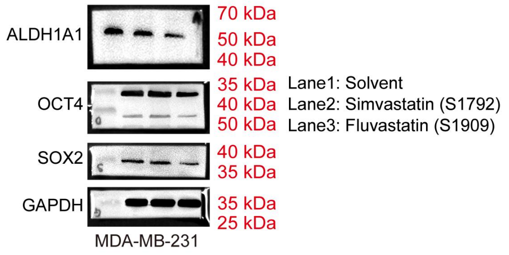

Supplementary Figure 4-5

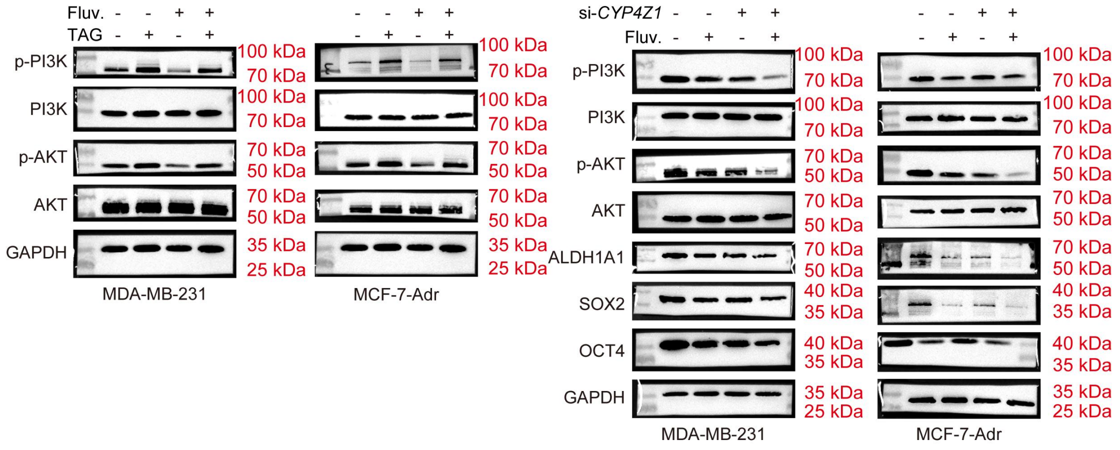

Supplementary Figure 7,9

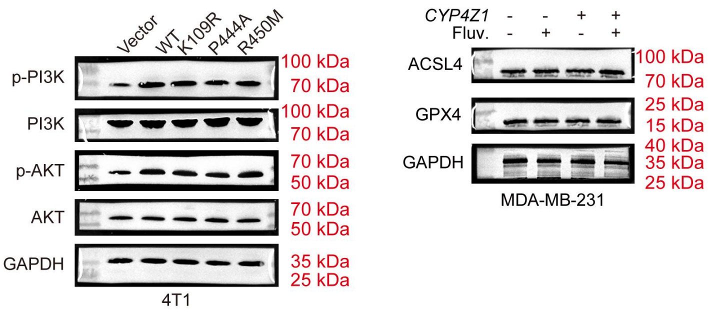

Supplementary Figure 13. Unedited western blots images, related to Supplementary Figure 1, 4, 5, 7, 9

Supplementary Tables

Supplementary Table 1. FDA-approved lipid-regulating drugs library.

Cat	Name	CAS number
S1655	Ezetimibe	163222-33-1
S1759	Pitavastatin calcium	147526-32-7
S1792	Simvastatin	79902-63-9
S1794	Fenofibrate	49562-28-9
S1806	Acipimox	51037-30-0
S1820	Clofibrate	637-07-0
S1909	Fluvastatin Sodium	93957-55-2
S2061	Lovastatin	75330-75-5
S2077	Atorvastatin Calcium	134523-03-8
S2169	Rosuvastatin calcium	147098-20-2
S2664	Clinofibrate	30299-08-2
S2665	Ciprofibrate	52214-84-3
S3036	Pravastatin sodium	81131-70-6
S4159	Bezafibrate	41859-67-0
S4223	Mevastatin	73573-88-3
S4264	Etofibrate	31637-97-5
S4527	Fenofibric acid	42017-89-0
S5713	Pravastatin	81093-37-0
S5715	Atorvastatin	134523-00-5
S5812	Choline Fenofibrate	856676-23-8
S6466	Eicosapentaenoic acid ethyl ester	86227-47-6
S7635	Lomitapide	182431-12-5
S7953	ETC-1002	738606-46-7

Supplementary Table 2. Sequences of primers for CYP4Z1 single-point mutation.

Name	Sequence (5'-3')
K109R-Forward	AGATCCCAGAAGTGCTGTTAGCCACAAAATCC
K109R-Reverse	CAGCACTTCTGGGATCTTGTCTTTTCAGGAGA
P444A-Forward	GCCTTCATAGCATTCTCAGCTGGATTAAGGAACTG
P444A-Reverse	GAGAATGcTATGAAGGCATAGGGATGTATTTTTT
R450M-Forward	AATGAACTGCATTGGGCAGCATTTTGCCATAA
R450M-Reverse	GCCCAATGCAGTTCTTTAATCCAGCTGAGAATGGTATGAA

Supplementary Table 3. Antibodies used in this study.

Antigens	Dilutions	Manufacturer	Cat No.
GAPDH	For WB, 1:50000	Proteintech	60004-1-Ig
SOX2	For WB, 1:500	Proteintech	11064-1-AP
ALDH1A1	For WB, 1:5000; for IF, 1:100	Proteintech	60171-1-Ig
OCT3/4	For WB, 1:5000	Wanleibio	WL01728
CYP4Z1	For WB, 1:1000	Invitrogen	PA5-101320
Ki67	For IF, 1:100	Proteintech	66555-6-Ig
AKT	For WB, 1:2000	Huaan Biotechnology	HA721870
p-AKT	For WB, 1:2000	Huaan Biotechnology	ET1607-73
PI3K	For WB, 1:1000	Huaan Biotechnology	HA722522
p-PI3K	For WB, 1:1000	Huaan Biotechnology	HA721672
ACSL4	For WB, 1:2000	Huaan Biotechnology	ET7111-43
GPX4	For WB, 1:2000	Huaan Biotechnology	ET1706-45
CD24-PE (anti-mouse)	For FC	Biolegend	114308
CD24-PE (anti-human)	For FC	Biolegend	382610
CD44-APC	For FC	Biolegend	163604
CD29-APC	For FC	Biolegend	102216
Gr-1-FITC	For FC	Biolegend	108406
Sca1-PE	For FC	Biolegend	108108
B220-FITC	For FC	Biolegend	103206
CD34-PE/Cyanine7	For FC	Biolegend	128618
IgM-FITC	For FC	Biolegend	406506
CD127-FITC	For FC	Biolegend	158208
CD3ε-FITC	For FC	Biolegend	152304
Ter119-FITC	For FC	Biolegend	116206
c-Kit-APC	For FC	Biolegend	135108
CD19-FITC	For FC	Biolegend	152404
CD48-PerCP/Cyanine5.5	For FC	Biolegend	103422
CD150-Brilliant Violet 421™	For FC	Biolegend	115926
CD16/32-Brilliant Violet 510™	For FC	Biolegend	156625
