# Supplementary material for: Fluvastatin suppresses breast cancer initiation and progression via targeting CYP4Z1
Source: Commun Biol. 2026 Jan 12;9:254. doi: 10.1038/s42003-026-09532-y (PMC12910054; doi:10.1038/s42003-026-09532-y)
Supplement: Supplementary file 3 — Description of additional supplementary [file 42003_2026_9532_MOESM3_ESM.pdf]

Description of additional supplementary

File Name: Supplementary Data.

Description : The numerical source data for the graphs is found in supplementary data.
